# Mitochondrial Haplogroup T Is Associated with Obesity in Austrian Juveniles and Adults

**DOI:** 10.1371/journal.pone.0135622

**Published:** 2015-08-31

**Authors:** Sabine Ebner, Harald Mangge, Helmut Langhof, Martin Halle, Monika Siegrist, Elmar Aigner, Katharina Paulmichl, Bernhard Paulweber, Christian Datz, Wolfgang Sperl, Barbara Kofler, Daniel Weghuber

**Affiliations:** 1 Department of Pediatrics, Paracelsus Medical University, Salzburg, Austria; 2 Clinical Institute for Medical and Chemical Laboratory Diagnostics, Medical University Graz, Graz, Austria; 3 BioTechMed-Graz, Graz, Austria; 4 Klinik Schönsicht, Berchtesgaden, Germany; 5 Department of Prevention, Rehabilitation and Sports Medicine, Klinikum rechts der Isar, Technische Universität München, Munich, Germany; 6 Department of Internal Medicine, Paracelsus Medical University, Salzburg, Austria; 7 Obesity Research Unit, Paracelsus Medical University and Salzburger Landeskliniken, Salzburg, Austria; 8 Department of Internal Medicine, General Hospital Oberndorf, Oberndorf, Austria; 9 DZHK (German Centre for Cardiovascular Research), partner site Munich Heart Alliance, Munich, Germany; 10 Else Kröner-Fresenius-Zentrum, Klinikum rechts der Isar, Munich, Germany; Ben-Gurion University of the Negev, ISRAEL

## Abstract

**Background:**

Recent publications have reported contradictory data regarding mitochondrial DNA (mtDNA) variation and its association with body mass index. The aim of the present study was to compare the frequencies of mtDNA haplogroups as well as control region (CR) polymorphisms of obese juveniles (n = 248) and obese adults (n = 1003) versus normal weight controls (n_juvenile_ = 266, n_adults_ = 595) in a well-defined, ethnically homogenous, age-matched comparative cohort of Austrian Caucasians.

**Methodology and Principal Findings:**

Using SNP analysis and DNA sequencing, we identified the nine major European mitochondrial haplogroups and CR polymorphisms. Of these, only the T haplogroup frequency was increased in the juvenile obese cohort versus the control subjects [11.7% in obese vs. 6.4% in controls], although statistical significance was lost after adjustment for sex and age. Similar data were observed in a local adult cohort, in which haplogroup T was found at a significantly higher frequency in the overweight and obese subjects than in the normal weight group [9.7% vs. 6.2%, p = 0.012, adjusted for sex and age]. When all obese subjects were considered together, the difference in the frequency of haplogroup T was even more clearly seen [10.1% vs. 6.3%, p = 0.002, OR (95% CI) 1.71 (1.2–2.4), adjusted for sex and age]. The frequencies of the T haplogroup-linked CR polymorphisms C16294T and the C16296T were found to be elevated in both the juvenile and the adult obese cohort compared to the controls. Nevertheless, no mtDNA haplogroup or CR polymorphism was robustly associated with any of several investigated metabolic and cardiovascular parameters (e.g., blood pressure, blood glucose concentration, triglycerides, cholesterol) in all obese subjects.

**Conclusions and Significance:**

By investigation of this large ethnically and geographically homogenous cohort of Middle European Caucasians, only mtDNA haplogroup T was identified as an obesity risk factor.

## Introduction

The heritability of obesity has long been appreciated. Early studies focused on monogenic and syndromal forms of extreme obesity, while subsequent genome-wide association studies (GWAS) of common genetic variants have identified novel loci. However, the common variants uncovered in the latter studies were found to have modest effects (per-allele odds ratios between 1.2 and 1.5), and the proportion of variability explained by the GWAS-identified loci to date remains relatively small (<10%) [1]. The heritability of obesity-related phenotypes may have been overestimated, as the effects of a shared environment are difficult to separate from inherited influences. Thus, several approaches are being used to explain the missing genetic heritability.

Given the essential function of mitochondria in energy metabolism, it seems plausible that mitochondrial dysfunction could play a role in obesity and its associated comorbidities [2–4]. A study of children with a suspected disorder of oxidative phosphorylation (OXPHOS) found a significant correlation between mitochondrial energy production and age-related body mass index (BMI) [4]. A Finnish study of identical twins who were either concordant or discordant for obesity showed that, independent of genetic factors, obesity was associated with poor fitness, low insulin sensitivity, and decreased transcript levels of genes involved in mitochondrial OXPHOS pathways [5]. Only a few studies have used large population samples to test the hypothesis that genetic variants in mtDNA might contribute to susceptibility to obesity, but they yielded conflicting results [6–9] (Table 1). It has long been recognized that certain morbidities are more prevalent in specific racial/ethnic populations [10–12]. Recently, striking geographic variation was shown for mtDNA [13, 14]. Thus, the quality of the sampling procedure is crucial to ensure the validity of the qualitative and quantitative results obtained from analysis of mtDNA.

**Table 1:** Literature reports of associations between mtDNA variants and obesity in different ethnicities.

Reference	Population studied	Homogenous population sample in regard to mtDNA haplogroups	Number of samples	Analyzed mtDNA variants/haplogroups	Results
Yang et al. 2011 [6]	Caucasians of Northern European origin living in Midwestern US	YES	2286 adults	72 mtDNA SNPs; 9 common European haplogroups	Haplogroup X, mt4823 and mt8873 associated with lower BMI and reduced body fat mass
Grant et al. 2011 [7]	European-Americans	NO	1080 obese children, 2500 lean children	138 mtDNA SNPs (including 19 haplogroup specific SNPs and 19 SNPs located in the D-loop)	No association with obesity for any SNP in both ethnicities and no difference in heteroplasmy
	African-Americans		1479 obese children, 1575 lean children		
Knoll et al. 2014 [8]	Discovery GWAS sample, participants from Germany and France, no information about ethnicity available	NO	1158 obese children and adolescents, 453 adult controls	35 mtDNA SNPs	Association with obesity found for G8994A; haplogroup W nominally overrepresented in the controls
	Confirmation GWAS sample, population-based, all residents from Southern, Northern and Northeastern Germany		1697 obese adults, 2373 adult controls		No association with obesity for any SNP or haplogroup
				D-loop polymorphisms in 192 extremely obese children and 192 lean adults (mainly originating from the discovery GWAS sample)	C16292T and C16189T associated with obesity
Nardelli et al. 2013 [9]	Caucasians from Southern Italy	YES	500 obese adults, 216 adult controls	9 common European haplogroups	Frequency of haplogroup T higher, J lower in obese; T haplogroup was correlated to the degree of obesity; No association of haplogroups and the tested clinical/biochemical variables

Whether mtDNA haplogroups contribute to obese phenotypes remains an unanswered question. To address this issue, the present study critically examines mtDNA haplogroups as well as control region (CR) polymorphisms in a large, well-defined, age-matched comparative cohort study including obese and normal weight subjects from an Austrian juvenile obese cohort (STYJOBS/EDECTA [15]), a local Austrian juvenile normal-weight cohort (URSPRUNG, [16]), and an adult Austrian confirmation cohort (SAPHIR, [17]).

## Materials and Methods

### Patients and control subjects

A total of 514 Middle European Caucasian juveniles (age<21 years) originating from two study populations was analyzed (Table 2). Siblings and subjects with other than Middle European ancestry were excluded from the study. Additionally, 1598 adults were included in our data analysis.

**Table 2:** Characteristics of the study populations.

	STYJOBS/	URSPRUNG^3^	SAPHIR^4^	SAPHIR^4^
	EDECTA^2^		obese	controls
	n = 248	n = 266	n = 1003	n = 595
Mean (SD^1^) age (years)	12.9 (3.1)	16.6 (1.7)	51.9 (6.0)	51.5 (6.2)
Male (%)	43.5	71.1	68.4	56.3
BMI (SD^1^) kg/m^2^	30.3 (6.1)	20.9 (1.9)	29.1 (3.5)	22.9 (1.6)

1 SD: standard deviation

2 Juvenile obese cohort 1

3 Juvenile control cohort

4 Adult cohort

#### 1. Juvenile cohort (STYJOBS/EDECTA)

DNA samples from 248 obese juveniles were obtained from the prospective, observational study STYrian Juvenile Obesity Study/Early DEteCTion of Arteriosclerosis [15, 18] collected at the Medical University Graz, Austria. The inclusion criteria for the overweight (obese) subjects was BMI>90^th^ percentile if below 18 years of age [19], and BMI>25 kg/m^2^ if above 18 years of age. Persons with endocrine, infectious, inflammatory or any other chronic disease were excluded from the study.

#### 2. Juvenile control cohort (URSPRUNG)

DNA samples from age-matched normal-weight controls were obtained from 266 students attending the Secondary School for Agriculture and Environmental Technology, Elixhausen, Salzburg [16]. The inclusion criterion for control subjects was BMI≤85th percentile [19].

#### 3. Adult cohort (SAPHIR)

As a confirmation sample, we recalculated the data of 1598 unrelated adult individuals recruited for the Salzburg Atherosclerosis Prevention Program [17]. Data on mitochondrial haplogroup analysis and CR polymorphisms were taken from previous studies [20–22]. For comparison of the frequencies of mtDNA haplogroups and CR polymorphisms, the SAPHIR cohort was divided into a normal-weight group (BMI≤25 kg/m^2^) and an overweight and obese group (BMI>25 kg/m^2^).

### Ethics Statement

The study was conducted according to the Austrian Gene Technology Act and complied with the Declaration of Helsinki in its revised version of 2013. All adult participants gave written informed consent before entering the study, and parental consent was obtained for juveniles.

The STYJOBS/EDECTA program is registered at ClinicalTrials.gov, Identifier NCT00482924. The URSPRUNG study, conducted as a school-based health survey project, was approved by the Austrian Ministery of Education, Science and Culture (Palais Starhemberg, Minoritenplatz, Vienna, Austria). The SAPHIR program was approved by the Local Province of Salzburg Ethics Committee ("Ethikkommission für das Bundesland Salzburg; Amt der Salzburger Landesregierung, Abteilung 9 Gesundheit und Sport").

### Mitochondrial DNA analysis

For the juvenile obese group (STYJOBS/EDECTA) and the adult SAPHIR cohort, a hierarchical system for mtDNA haplogrouping that combines multiplex PCR amplification, multiplex single-base primer extension, and capillary-based electrophoretic separation was used to assess the most common European haplogroups (H, U, J, T, K, I, V, W and X) as described previously [23]. Haplogroups that could not be assigned to one of the nine major European haplogroups by their single nucleotide polymorphism (SNP) combination were designated as ‘others’. SNP analysis for haplogrouping of the juvenile control group (URSPRUNG) was performed at Sequenom GmbH Hamburg, Germany using the MALDI-TOF mass spectrometry-based iPLEX Gold assay [24].

CR sequences were generated by direct DNA sequencing between nucleotide positions (np) 16024 and 526 for all juvenile groups (STYJOBS/EDECTAand URSPRUNG) and between np 16145 and 509 for the adult SAPHIR cohort. Polymerase chain reaction and sequencing was performed as described previously [25]. Data were analyzed with Chromas software 1.56 (Technelysium, Tewantin, Australia) and alignment was conducted with Blast 2 software (bl2seq) (http://blast.ncbi.nlm.nih.gov/Blast.cgi). The Cambridge Revised sequence was used as a reference (GenBank accession number J01415).

### Statistical analysis

Frequencies of all mitochondrial haplogroups and CR polymorphisms were tested for independency for the disease using a non-parametric Mann-Whitney-U test as appropriate. Only haplogroups and polymorphisms with a frequency ≥5% in either the obese or the control group were subjected to further statistical analysis. P-values were corrected for multiple comparison using Bonferroni analysis (required level of significance = 0.05/number of comparisons), leading to a new required significance level of <0.005 for analysis of mitochondrial haplogroups [number of comparisons = 10]. The respective level of significance for analysis of CR polymorphisms was <0.0003 for the STYJOBS/EDECTA juvenile obese group [number of comparisons = 192] and <0.0002 for the SAPHIR adult obese group [number of comparisons = 333]. Additionally, logistic regression analysis was applied to adjust for possible confounders (sex and age).

The following clinical and biochemical variables were analyzed in the juvenile obese group for association with specific mtDNA haplogroups or CR polymorphisms: BMI, BMI standard deviation score (BMI sds), diastolic blood pressure, systolic blood pressure, blood glucose concentration, insulin resistance (HOMA-Homeostasis Model Assessment), triglycerides (TGs), low-density lipoprotein-cholesterol (LDL), high-density lipoprotein-cholesterol (HDL), waist-to-hip ratio (WHR), body fat percentage, birthweight, BMI of mother, fasting insulin, ultra-sensitive C-reactive protein (CRP), adiponectin, intima-media thickness (IMT), family history of diabetes mellitus type 2, family history of hypertension, family history of myocardial infarction (MI) and family history of stroke.

For the SAPHIR study group the following variables were available: BMI, blood glucose concentration, fasting insulin, insulin resistance (HOMA-Homeostasis Model Assessment), waist circumference (WC), hip circumference (HC), visceral adipose tissue (VAT), subcutaneous adipose tissue (SAT), body fat percentage, lean body mass (LBM), ultra-sensitive C-reactive protein (CRP) and adiponectin.

ANOVA was used to compare metabolic parameters in the different mtDNA haplogroups. To test for an association between clinical and biochemical parameters and presence of a specific mtDNA haplogroup or CR polymorphism, an independent sample t-test (for normally distributed variables) or a non-parametric Mann-Whitney U-test was performed. The Kolmogorov-Smirnov test was used to check for normality.

All analyses were performed using PASW 18.0 (SPSS GmbH, Germany).

## Results

The clinical characteristics of all study groups are shown in Table 2.

### mtDNA haplogroup T is overrepresented in overweight/obese children and overweight/obese adults

#### 1. Juvenile cohort (STYJOBS/EDECTA)

The frequency of the T haplogroup was higher in the obese group compared to the URSPRUNG age-matched control group [11.7% in obese vs. 6.4% in controls, p = 0.036] (Table 3), however not reaching the required evel of significance for multiple testing of <0.005. The frequencies of all other major mitochondrial haplogroups were not different.

**Table 3:** Frequencies (%) of Caucasian mitochondrial haplogroups in juvenile obesity cases and controls.

mtDNA haplogroup	Frequency (%) in STYJOBS/ EDECTA^3^	n^1^	Frequency (%) in URSPRUNG^4^	n^1^
H	42.4	105	44.7	119
U	16.9	42	14.7	39
J	9.3	23	12.0	32
T	11.7	29	6.4	17
K	2.8	7	3.8	10
W	0.0	0	1.1	3
V	3.6	9	3.0	8
I	1.2	3	1.5	4
X	2.8	7	3.4	9
Others^2^	9.3	23	9.4	25

n^1^ = Number of individuals with respective mtDNA haplogroup.

2Haplogroups that could not be assigned to one of the nine major European haplogroups by the SNP combination.

3Juvenile obese cohort 1

^4^Juvenile control cohort

#### 2. Adult cohort (SAPHIR)

In the overweight and obese adult subjects, haplogroup T was again found at a higher frequency than in the normal weight group (Table 4) [9.7% vs. 6.2%, p = 0.016]. None of the other haplogroups showed a possible association with obesity.

**Table 4:** Frequencies (%) of Caucasian mitochondrial haplogroups in adult obesity cases and controls.

mtDNA haplogroup	Frequency (%) in overweight/obese SAPHIR^3^ (BMI>25)	n^1^	Frequency (%) in normal weight SAPHIR^3^ (BMI≤25)	n^1^
H	44.0	441	44.0	262
U	14.2	142	17.0	101
J	10.8	108	11.8	70
T	9.7	97	6.2	37
K	5.3	53	5.7	34
W	2.2	22	1.8	11
V	1.7	17	1.7	10
I	0.9	9	1.0	6
X	1.8	18	0.8	5
Others^2^	9.6	96	9.9	59

n^1^ = Number of individuals with respective mtDNA haplogroup.

2 Haplogroups that could not be assigned to one of the nine major European haplogroups by the SNP combination.

3 Adult cohort

#### 3. All juvenile and adult obese subjects

When all (n = 1251) were compared to the controls of all age groups (n = 861), the higher frequency of haplogroup T in the obese subjects was significant and remained robust after adjustment for sex and age and correction for multiple testing [10.1% vs. 6.3%, p = 0.002, OR (95% CI) 1.68 (1.2–2.3)].

### CR polymorphisms in obese/overweight subjects

mtDNA D-loop variants of all 514 juvenile samples were investigated by direct sequencing of a 1066-bp fragment between np 16024 and 526. In 1598 adult samples, sequences were analyzed between np 16145 and 509. Only CR polymorphisms with a frequency ≥5% in either controls or cases were deemed to be relevant and analyzed further.

#### 1. Juvenile cohort (STYJOBS/EDECTA)

In the Austrian juvenile obese cohort, we detected 192 homoplasmic polymorphisms (Table A in S1 File), six of which were not listed in either Genbank (www.ncbi.nlm.nih.gov/genbank), Phylotree (www.phylotree.org) or MITOMAP (www.mitomap.org/MITOMAP) [26]. In detail, we found 175 single base-pair exchanges, six single base-pair deletions and three single base-pair insertions, compared to the revised Cambridge Reference Sequence. At position 302 both single C-insertions and multiple C-insertions (2–4 Cs) were detected. Furthermore, we detected a TC-insertion at position 310. CA- and CACA-insertions were observed at position 513, whereas CA-deletions occurred at positions 514 and 515.

Thirty-nine of the 192 homoplasmic polymorphisms detected were found at a frequency ≥5% in either the obese or the control (URSPRUNG) subjects (Table 5) and were subjected to further statistical analysis. The frequencies of the C16294T, C16296T, G16526A, and A263G substitution were higher in the STYJOBS/EDECTA group than in the controls, whereas the T146C substitution was detected at a lower frequency in obese subjects. However, statistical significance was lost after correction for multiple testing.

**Table 5:** Frequencies (%) of CR polymorphisms higher than 5% in either juvenile obese Austrians (STYJOBS/EDECTA) or juvenile controls (URSPRUNG) and odds ratios (OR) for the association between genetic variation and disease state.

mtDNA CR polymorphism	Frequency in STYJOBS/EDCTA (%)	n^1^	Frequency in URSPRUNG (%)	n^1^	p-value^2^	OR^3^ (95%CI^4^)	p-value^5^	OR (95%CI)^5^
C16069T	9.3	23	11.3	30	0.456			
T16093C	3.6	9	6.4	17	0.154			
T16126C	21.8	54	20.7	55	0.761			
G16145A	2.4	6	5.3	14	0.096			
A16183C	7.7	19	8.3	22	0.799			
T16189C	19.8	49	19.5	52	0.952			
Uninterrupted poly-C tract	14.9	37	15.8	42	0.785			
C16192T	6.9	17	5.6	15	0.569			
C16223T	7.3	18	8.6	23	0.562			
T16224C	3.2	8	6.4	17	0.096			
C16256T	5.2	13	4.9	13	0.855			
C16261T	3.6	9	6.8	18	0.111			
C16270T	10.5	26	9.8	26	0.790			
C16294T	12.9	32	6.4	17	0.012	2.17 (1.2–4.0)	0.052	2.18 (1.0–4.8)
C16296T	6.5	16	1.9	5	0.009	3.60 (1.3–10.0)	0.278	1.92 (0.6–6.3)
T16298C	6.9	17	4.1	11	0.175			
T16304C	10.9	27	6.8	18	0.099			
T16311C	10.1	25	15.0	40	0.091			
T16356C	7.3	18	6.4	17	0.697			
T16362C	11.3	28	8.3	22	0.249			
T16519C	60.1	149	58.3	155	0.677			
G16526A	6.5	16	1.5	4	0.004	4.52 (1.5–13.7)	0.028	5.10 (1.2–21.8)
A73G	52.4	130	51.9	138	0.903			
T146C	5.2	13	11.3	30	0.014	0.44 (0.2–0.9)	0.079	0.46 (0.2–1.1)
C150T	9.7	24	12.0	32	0.393			
T152C	19.4	48	23.7	63	0.234			
G185A	5.2	13	5.6	15	0.843			
T195C	17.7	44	19.2	51	0.676			
G228A	8.1	20	5.6	15	0.276			
A263G	100.0	248	97.7	260	0.017	0.98 (0.96–1.0)		
C295T	8.1	20	12.4	33	0.106			
A302C-Ins	42.3	105	43.6	116	0.772			
A302CC-Ins	14.1	35	12.0	32	0.484			
T310C-Ins	98.0	243	95.5	254	0.114			
C456T	5.6	14	4.5	12	0.427			
C462T	6.9	17	6.8	18	0.968			
T489C	9.7	24	12.4	33	0.325			
G513CA-Ins	6.5	16	4.5	12	0.333			
C514Del	11.7	29	9.0	24	0.320			
A515Del	11.7	29	9.0	24	0.320			

^1^n: number of individuals with the respective polymorphism.

2 p-value: derived from Mann-Whitney-U test.

3 OR: Odds Ratio

4 CI: Confidence Interval

5 adjusted for sex and age

#### 2. Adult cohort (SAPHIR)

Among the overweight and obese subjects of the SAPHIR study, 333 homoplasmic polymorphisms were detected (Table B in S1 File). Seventeen of these polymorphisms were not listed in either GeneBank, Phylotree or MITOMAP. We discovered 314 single base-pair exchanges, ten single base-pair deletions and five single base-pair insertions, compared to the revised Cambridge Reference Sequence. At position 302, again multiple C-insertions (2–3 Cs) could be found. Furthermore, we detected a TC- and a TCC-insertion at position 310.

Twenty-seven of the 333 homoplasmic polymorphisms were present at a frequency ≥5% in either the overweight and obese SAPHIR subjects or the normal weight SAPHIR subjects (Table 6) and were subjected to further statistical analysis. The frequency of the T haplogroup marker C16294T was higher [p = 0.008, OR (95% CI) 1.68 (1.1–2.5), after adjustment for sex and age]therefore nearly reaching significance after correction for multiple testing. In contrast, the frequency of the G228A substitution was lower in the overweight and obese SAPHIR subjects compared to the normal weight controls [p = 0.042, OR (95% CI) 0.65 (0.4–1.0), after adjustment for sex and age].

**Table 6:** Frequencies (%) of CR polymorphisms higher than 5% in either overweight and obese or normal weight adults (both SAPHIR cohort) and odds ratios (OR) for the association between genetic variation and disease state.

mtDNA CR polymorphism	Frequency in overweight and obese SAPHIR (%)	n^1^	Frequency in lean and normal weight SAPHIR (%)	n^1^	p-value^2^	OR^3^ (95%CI^4^)	p-value^5^	OR (95%CI)^5^
T16189C	11.7	117	12.3	73	0.719			
Uninterrupted poly-C tract	9.2	92	9.4	56	0.873			
C16192T	5.4	54	7.1	42	0.173			
C16223T	6.1	61	5.9	35	0.871			
T16224C	7.2	72	8.7	52	0.260			
C16256T	6.3	63	5.9	35	0.748			
C16270T	7.7	77	8.1	48	0.776			
C16294T	10.6	106	6.7	40	0.010	1.64 (1.1–2.4)	0.008	1.68 (1.1–2.5)
C16296T	6.9	69	4.7	28	0.079	1.50 (1.0–2.3)	0.098	1.47 (0.9–2.3)
T16304C	7.9	79	7.7	46	0.917			
T16311C	13.1	131	15.1	90	0.248			
T16356C	3.7	37	5.0	30	0.192			
T16362C	6.9	69	6.7	40	0.904			
T16519C	66.8	670	64.7	385	0.393			
A73G	53.9	541	54.8	326	0.741			
T146C	9.2	92	9.2	55	0.962			
C150T	10.6	106	12.4	74	0.254			
T152C	22.8	229	22.5	134	0.886			
G185A	5.5	55	6.1	36	0.637			
T195C	16.9	170	17.3	103	0.853			
G228A	5.3	53	7.4	44	0.088	0.70 (0.5–1.1)	0.042	0.65 (0.4–1.0)
A263G	98.9	992	98.7	587	0.659			
C295T	10.1	101	10.8	64	0.667			
A302C-Ins	39.2	393	36.5	217	0.281			
A302CC-Ins	11.7	117	11.8	70	0.952			
T310C-Ins	97.3	976	96.1	572	0.193			
C462T	8.1	81	8.7	52	0.643			
T489C	11.3	113	11.9	71	0.687			

^1^n: number of individuals with the respective polymorphism.

2 p-value: derived from Mann-Whitney-U test.

3 OR: Odds Ratio

4 CI: Confidence Interval

5 adjusted for sex and age

### Analysis of clinical and biochemical parameters

General and biochemical characteristics of the study populations are shown in Table C in S1 File. No association of relevant metabolic and cardiovascular parameters with a specific mtDNA haplogroup or CR polymorphism in any of the obese groups could be detected.

## Discussion

In the past five years, several studies have reported contradictory findings regarding the contribution that the mitochondrial genotype may make to pediatric and adult obesity (Table 1).

Here we report our results of the first age-matched comparative cohort study of juvenile obesity to analyze mtDNA haplogroups as well as CR polymorphisms. As the quality of the sampling procedure can have an impact on the qualitative and quantitative results obtained from the analysis of mtDNA [27], and because mtDNA shows striking regional variation [14, 28, 29], we placed our emphasis on a juvenile study group as homogeneous as possible. Furthermore, mtDNA haplogroups and CR polymorphisms as possible risk factors for obesity were analyzed in a well-characterized adult study group.

Haplogroup T was overrepresented in the obese juveniles, although the association was not significant after adjustment for multiple testing [p = 0.036]. However, this tendency of haplogroup T to be more frequent in obese subjects is supported by data from the Austrian SAPHIR adult study group: the frequency of haplogroup T was higher in the adult overweight and obese subjects than in the normal weight controls [p = 0.016], however not reaching the required level of significance for multiple testing of <0.005. When all juvenile and adult obese subjects (n = 1251) were compared to the controls of all age groups (n = 861), a potential association of haplogroup T and obesity was even more pronounced [p = 0.002, OR (95% CI) 1.68 (1.2–2.3)].

The presented data are in agreement with the results of Nardelli et al. [9], who analyzed an ethnically and geographically homogenous (Italian) study group (obese patients n = 500; controls n = 216) similar to that in our study. In addition to finding an elevation of the T haplogroup frequency in their obese subjects, Nardelli et al. also observed a tendency for underrepresentation of haplogroup J in the same obese cohort. This is an interesting observation, as mitochondrial haplogroup J and T form a cluster with shared polymorphisms (e.g. mtDNA T4216C) and were formerly described as sister haplogroups with a common root [30]. Our results contradict those of Knoll et al. [8], who found no association of mtDNA haplogroups and obesity. However, it should be noted that the cases and control subjects of the Knoll study were either of French or German ancestry [31]; therefore, differences of geographic origin might account for the different results of the two studies. Indeed, as early as 1996, Torroni et al. pointed out that certain variants and haplogroups can be common in certain populations and absent in other ethnic groups [28]. Consequently, those authors postulated that mtDNA disease studies require control populations that are accurately ethnically matched to the background of the patients. This was further supported by studies in Ashkenazi Jewish individuals demonstrating the need to take population substructure into account when designing association studies [29].

Previously, haplotype T was reported to be a risk factor for multifactorial disorders such as coronary artery disease and diabetic retinopathy [22]. Furthermore, the T haplogroup has been shown to be negatively associated with elite endurance training [32]. There is evidence that different mtDNA haplogroups are associated with subtle differences in OXPHOS capacity and generation of reactive oxygen species (ROS), which may have functional consequences [33–36]. Our data suggest that there is a population-specific association of mtDNA haplogroup T with obesity in Middle European Caucasians, although it still has to be elucidated whether there is a functional association between haplogoup T and the phenotype.

Although most mitochondrial polymorphisms are believed to be neutral, population-specific mtDNAs may be functionally different and exert varying influences on the outcome of a disease [13]. Different combinations of certain mtDNA variants might, in a synergistic way, influence the susceptibility of certain haplogroups to disease by causing differences in energy production. Variants of the non-coding CR of mtDNA are of particular interest because they may affect mtDNA transcription and replication and therefore may contribute to the etiology of a disease. Herein, we evaluated CR polymorphisms with a frequency ≥5% and their possible role as risk factors for juveniles and adults. Most interestingly, the frequency of the C16294T mutation was elevated in the two obese groups compared to the controls. This can be explained by the fact that C16294T is a haplotype T marker and therefore is linked to an increased frequency of haplogroup T in the obese subjects. Similarly, the subhaplogroup T2 marker C16296T was detected more frequently in our juvenile obese cohort but not in the adult obese cohort.

Knoll et al. reported a higher frequency of the polymorphism T16189C among extremely obese cases (17% vs. 9%) [8]. This variant would give rise to an uninterrupted poly-C tract from np 16184 to 16193 and possibly lead to heteroplasmic length variation of different mtDNA molecules within the same person [37]. The polymorphism T16189C has been described to be associated with other multifactorial diseases, including type 2 diabetes mellitus [20, 38], coronary artery disease [20] and metabolic syndrome [39, 40]. In contrast to the results of Knoll et al., the 16189 variant has been reported to be associated with thinness in 161 Australian mothers and their 20-yr-old offspring. [41]. In the present study, we did not detect an association of the 16189 variant with obesity or BMI. On the contrary, we even detected a trend of lower T16189C frequencies in obese subjects compared to controls.

We further looked for potential correlations of certain mtDNA haplogroups or CR polymorphisms with obesity-relevant metabolic and cardiovascular parameters. However, no mtDNA variant was robustly associated with any of the investigated metabolic and cardiovascular parameters in both the obese juvenile cohort and the adult obese cohort. Nor could we confirm the results of Yang et al., who reported an association of haplogroup X with lower BMI and body fat mass in a sample of unrelated Northern European Caucasians [6]. Saxena and coworkers, as well, did not find any significant association of common mtDNA variants (except CR polymorphisms) with metabolic phenotypes in five analyzed ethnicities [42]. Due to the lack of association with obesity-related clinical markers it is tempting to hypothesize directionality from the mitochondrial variants to the obese phenotype, rather than the reverse causal directionality.

There are study limitations that need to be acknowledged. Firstly, since the data of the adult study group were taken from a previous study, our study design was post-hoc. However, analyzing both the juvenile and the adult subjects together had the advantage to increase the number of samples as it is widely recognized that small sample sizes do have limited study power [43]. However, a potential predictive value of haplogroup T for weight gain and/or obesity-related morbidity warrants further evaluation in a longitudinal study design. Further, as mentioned above, geographic and ethnic homogeneity is key to mitochondrial genetic studies. Thus, the current study particularly strived to ensure regional and ethnical homogeneity.

In summary, our study found the frequencies of mitochondrial haplogroup T and a linked D-loop variant (C16294T) to be elevated in a juvenile obese cohort and an adult obese cohort of Middle European ancestry. However, the frequency of the subhaplogroup T2 marker C16296T was elevated only in the juvenile cohort, but not in the adult group. No association of certain mtDNA haplogroups or CR polymorphisms with relevant metabolic and cardiovascular parameters could be detected. In conclusion, our data suggest that there is a population-specific association of mtDNA haplogroup T with obesity in Middle European Caucasians.

## Supporting Information

S1 File Table A. CR polymorphisms of juvenile obese cohort 1 (STYJOBS/EDECTA). Table B. CR polymorphisms of the adult obese cohort (SAPHIR). Table C. General and biochemical characteristics of the study populations. (DOCX)
