# Supplementary material for: Mitochondrial Haplogroup T Is Associated with Obesity in Austrian Juveniles and Adults
Source: PLoS One. 2015 Aug 31;10(8):e0135622. doi: 10.1371/journal.pone.0135622 (PMC4556186; doi:10.1371/journal.pone.0135622)
Supplement: S1 File — (DOCX) [file pone.0135622.s001.docx]

## Supporting Information

**Table A.** CR polymorphisms of juvenile obesity cohort (STYJOBS/EDECTA).

| Polymorphism in mtDNA control region | Frequency (%) in STYJOBS/EDECTA | n^1^ |
| --- | --- | --- |
| A16037G | 0.4 | 1 |
| G16048A | 0.4 | 1 |
| A16051G | 2.8 | 7 |
| C16069T | 9.3 | 23 |
| A16081G | 0.4 | 1 |
| G16084A | 0.4 | 1 |
| T16086C | 0.4 | 1 |
| T16092C | 2.8 | 7 |
| T16093C | 3.6 | 9 |
| C16114A | 0.4 | 1 |
| T16126C | 21.8 | 54 |
| G16129A | 4.8 | 12 |
| G16129C | 2.0 | 5 |
| T16140C | 1.2 | 3 |
| T16144C | 0.4 | 1 |
| G16145A | 2.4 | 6 |
| C16147T | 0.4 | 1 |
| G16153A | 2.0 | 5 |
| A16162G | 2.0 | 5 |
| A16163G | 2.8 | 7 |
| A16166G | 0.4 | 1 |
| C16168T | 0.4 | 1 |
| C16169T | 0.4 | 1 |
| A16170T | 0.4 | 1 |
| T16172C | 2.4 | 6 |
| C16174T | 0.8 | 2 |
| C16179T | 1.6 | 4 |
| A16182C | 3.2 | 8 |
| A16182G | 0.4 | 1 |
| A16183C | 7.7 | 19 |
| A16183A-del | 0.4 | 1 |
| C16184T | 0.4 | 1 |
| C16185T | 0.4 | 1 |
| C16186T | 3.6 | 9 |
| C16187T | 0.8 | 2 |
| C16188T | 0.8 | 2 |
| T16189A | 0.4 | 1 |
| T16189C | 19.8 | 49 |
| C16192T | 6.9 | 17 |
| G16213A | 1.2 | 3 |
| A16216G | 0.8 | 2 |
| T16217C | 0.8 | 2 |
| C16218T | 0.4 | 1 |
| A16220C | 0.4 | 1 |
| C16222T | 0.8 | 2 |
| C16223T | 7.3 | 18 |
| T16224C | 3.2 | 8 |
| A16230G | 0.4 | 1 |
| T16231C | 1.6 | 4 |
| A16235G | 0.8 | 2 |
| C16239A | 0.8 | 2 |
| C16239T | 1.2 | 3 |
| A16246T | 0.4 | 1 |
| C16248T | 1.2 | 3 |
| T16249C | 1.2 | 3 |
| G16255A | 0.4 | 1 |
| C16256G | 0.8 | 2 |
| C16256T | 5.2 | 13 |
| A16258C | 0.4 | 1 |
| A16258T | 0.4 | 1 |
| C16261T | 3.6 | 9 |
| C16264T | 0.4 | 1 |
| A16265G | 2.0 | 5 |
| C16266T | 0.8 | 2 |
| C16270T | 10.5 | 26 |
| G16274A | 1.6 | 4 |
| C16278T | 2.8 | 7 |
| C16287A | 0.8 | 2 |
| T16288C | 1.6 | 4 |
| C16291T | 4.8 | 12 |
| C16292T | 1.2 | 3 |
| A16293G | 1.2 | 3 |
| C16294T | 12.9 | 32 |
| C16295T | 0.4 | 1 |
| C16296T | 6.5 | 16 |
| T16297C | 0.4 | 1 |
| T16298C | 6.9 | 17 |
| A16300G | 0.8 | 2 |
| T16304C | 10.9 | 27 |
| T16311C | 10.1 | 25 |
| A16316G | 1.2 | 3 |
| G16319A | 0.4 | 1 |
| C16320T | 1.2 | 3 |
| C16327T | 0.4 | 1 |
| T16342C | 0.4 | 1 |
| A16343G | 0.8 | 2 |
| C16344T | 0.4 | 1 |
| G16346C | 0.4 | 1 |
| C16355T | 1.2 | 3 |
| T16356C | 7.3 | 18 |
| T16362C | 11.3 | 28 |
| C16363C-del^2^ | 0.4 | 1 |
| C16365T | 0.4 | 1 |
| G16390A | 0.4 | 1 |
| G16391A | 0.8 | 2 |
| G16398T^2^ | 0.4 | 1 |
| A16399G | 2.8 | 7 |
| T16443C^2^ | 0.4 | 1 |
| C16454T | 0.4 | 1 |
| G16456A | 0.4 | 1 |
| G16457T^2^ | 0.4 | 1 |
| A16463G | 0.4 | 1 |
| C16465T | 0.4 | 1 |
| G16474C | 0.4 | 1 |
| G16474T | 0.4 | 1 |
| A16476G^2^ | 0.4 | 1 |
| A16482G | 2.0 | 5 |
| T16519C | 60.1 | 149 |
| G16526A | 6.5 | 16 |
| C16527T | 0.4 | 1 |
| T16545C | 0.4 | 1 |
| C41T | 0.8 | 2 |
| C64T | 0.4 | 1 |
| T72A | 0.4 | 1 |
| T72C | 3.6 | 9 |
| T72G | 0.4 | 1 |
| A73G | 52.4 | 130 |
| A93G | 1.6 | 4 |
| G94A | 0.4 | 1 |
| C114T | 0.4 | 1 |
| G143A | 1.6 | 4 |
| T146C | 5.2 | 13 |
| C150T | 9.7 | 24 |
| C151T | 1.2 | 3 |
| T152C | 19.4 | 48 |
| T152T-del | 0.4 | 1 |
| A153G | 2.0 | 5 |
| T179A^2^ | 0.4 | 1 |
| C182T | 0.4 | 1 |
| G184C | 0.4 | 1 |
| G185A | 5.2 | 13 |
| G185T | 0.4 | 1 |
| C186A | 0.4 | 1 |
| A188G | 1.2 | 3 |
| A189G | 2.4 | 6 |
| C194T | 0.8 | 2 |
| T195C | 17.7 | 44 |
| T199C | 2.4 | 6 |
| A200G | 0.4 | 1 |
| G203A | 0.4 | 1 |
| T204C | 3.6 | 9 |
| G207A | 2.8 | 7 |
| A215G | 1.2 | 3 |
| T217C | 1.6 | 4 |
| G225A | 2.0 | 5 |
| T226C | 0.4 | 1 |
| A227G | 0.4 | 1 |
| G228A | 8.1 | 20 |
| A230G | 0.4 | 1 |
| A235G | 0.4 | 1 |
| T239C | 2.0 | 5 |
| A240G | 0.4 | 1 |
| C242T | 0.4 | 1 |
| G247A | 1.2 | 3 |
| T250C | 1.2 | 3 |
| A263G | 100.0 | 248 |
| C295A | 0.8 | 2 |
| C295T | 8.1 | 20 |
| A302C-ins | 42.3 | 105 |
| A302CC-ins | 14.1 | 35 |
| A302CCC-ins | 1.6 | 4 |
| A302CCCC-ins | 0.4 | 1 |
| T310C-ins | 98.0 | 243 |
| T310TC-ins | 1.2 | 3 |
| T319C | 1.2 | 3 |
| A335T^2^ | 0.4 | 1 |
| C340T | 0.4 | 1 |
| A350A-del^2^ | 0.4 | 1 |
| A385G | 0.4 | 1 |
| A390T | 0.4 | 1 |
| T393A^2^ | 0.4 | 1 |
| G417A | 0.4 | 1 |
| C437T | 0.4 | 1 |
| T452T-del | 0.4 | 1 |
| T452T-ins^2^ | 0.4 | 1 |
| C456T | 5.6 | 14 |
| C462T | 6.9 | 17 |
| T477C | 1.2 | 3 |
| T482C | 0.8 | 2 |
| T489C | 9.7 | 24 |
| T489G | 0.4 | 1 |
| C494C-del | 1.2 | 3 |
| C497T | 0.8 | 2 |
| G499A | 4.0 | 10 |
| C506A | 0.4 | 1 |
| A508G | 2.0 | 5 |
| A512G | 0.4 | 1 |
| G513A | 2.0 | 5 |
| G513CA-ins | 6.5 | 16 |
| G513CACA-ins | 0.8 | 2 |
| C514C-del | 11.7 | 29 |
| A515C-del | 11.7 | 29 |

^1^n = Number of individuals with the respective polymorphism

^2^Polymorphisms not listed in Genbank, MITOMAP or Phylotree

**Table B.** CR polymorphisms of the adult obesity cohort (SAPHIR).

| Polymorphism in mtDNA control region | Frequency (%) | n^1^ |
| --- | --- | --- |
| G16145A | 4.2 | 42 |
| C16147A | 0.3 | 3 |
| C16147T | 0.1 | 1 |
| C16148T | 0.6 | 6 |
| G16153A | 0.7 | 7 |
| T16154A | 0.2 | 2 |
| T16154C | 0.3 | 3 |
| A16162G | 2.6 | 26 |
| A16163G | 1.7 | 17 |
| A16163T | 0.2 | 2 |
| C16167T | 0.1 | 1 |
| C16168T | 0.2 | 2 |
| C16169T | 0.2 | 2 |
| A16170G | 0.1 | 1 |
| T16172C | 2.8 | 28 |
| C16173G | 0.1 | 1 |
| C16173T | 0.1 | 1 |
| C16174T | 0.1 | 1 |
| C16176A | 0.2 | 2 |
| C16176G | 0.2 | 2 |
| C16176T | 0.1 | 1 |
| C16179A | 0.2 | 2 |
| C16179G | 0.1 | 1 |
| C16179T | 0.9 | 9 |
| A16180C | 0.1 | 1 |
| A16180G | 0.1 | 1 |
| A16180T | 0.1 | 1 |
| A16182C | 0.4 | 4 |
| A16182G | 0.3 | 3 |
| A16182Del | 0.3 | 3 |
| A16183C | 1.0 | 10 |
| A16183G | 0.1 | 1 |
| A16183Del | 1.3 | 13 |
| C16185T | 0.3 | 3 |
| C16186T | 1.5 | 15 |
| C16188G | 0.1 | 1 |
| C16188T | 0.7 | 7 |
| T16189A | 0.3 | 3 |
| T16189C | 11.7 | 117 |
| C16192T | 5.4 | 54 |
| C16193T | 1.9 | 19 |
| T16195C | 0.4 | 1 |
| C16201T | 0.1 | 1 |
| A16207G | 0.4 | 1 |
| T16209C | 0.6 | 6 |
| A16212G | 0.4 | 4 |
| G16213A | 0.3 | 3 |
| C16214T | 0.1 | 1 |
| A16216G | 0.1 | 1 |
| A16219G | 0.1 | 1 |
| A16220T | 0.1 | 1 |
| C16221T | 1.0 | 10 |
| C16222T | 2.2 | 22 |
| C16223T | 6.1 | 61 |
| T16224C | 7.2 | 72 |
| T16231C | 2.3 | 23 |
| A16233G | 0.3 | 3 |
| C16234T | 0.5 | 5 |
| A16235G | 0.3 | 3 |
| C16239T | 1.7 | 17 |
| A16240G | 0.2 | 2 |
| A16241C | 0.1 | 1 |
| A16241G | 0.1 | 1 |
| C16245T | 0.1 | 1 |
| A16246T | 0.1 | 1 |
| C16248T | 0.9 | 9 |
| T16249C | 0.8 | 8 |
| G16255A | 0.1 | 1 |
| G16255T | 0.1 | 1 |
| C16256T | 6.3 | 63 |
| C16257T | 0.1 | 1 |
| A16258C | 0.5 | 5 |
| A16258G | 0.1 | 1 |
| A16258T | 0.6 | 6 |
| C16259G | 0.2 | 2 |
| C16259T | 0.1 | 1 |
| C16260T | 0.2 | 2 |
| C16261T | 3.9 | 39 |
| T16263C | 0.4 | 4 |
| C16264T | 0.1 | 1 |
| A16265C | 0.2 | 2 |
| A16265G | 0.5 | 5 |
| A16265T | 0.2 | 2 |
| C16266T | 0.5 | 5 |
| A16269G | 0.2 | 2 |
| C16270A | 0.1 | 1 |
| C16270G | 0.2 | 2 |
| C16270T | 7.7 | 77 |
| T16271C | 0.3 | 3 |
| G16274A | 0.8 | 8 |
| G16274T | 0.1 | 1 |
| C16278T | 3.0 | 30 |
| A16284G | 0.1 | 1 |
| C16286T | 0.1 | 1 |
| C16287A | 0.5 | 5 |
| C16287T | 0.3 | 3 |
| T16288C | 0.1 | 1 |
| C16290G | 0.1 | 1 |
| C16290T | 0.2 | 2 |
| C16291T | 1.9 | 19 |
| C16292T | 2.6 | 26 |
| A16293C | 0.1 | 1 |
| A16293G | 1.1 | 11 |
| C16294A | 0.2 | 2 |
| C16294T | 10.6 | 106 |
| C16295T | 0.4 | 4 |
| C16296T | 6.9 | 69 |
| T16297C | 0.1 | 1 |
| T16298C | 4.7 | 47 |
| A16299G | 0.8 | 8 |
| A16300G | 0.6 | 6 |
| C16301T | 0.1 | 1 |
| A16302G | 0.1 | 1 |
| T16304C | 7.9 | 79 |
| A16309G | 0.4 | 4 |
| G16310A | 0.1 | 1 |
| G16310T | 0.1 | 1 |
| T16311C | 13.1 | 131 |
| A16316G | 0.9 | 9 |
| A16318C | 0.2 | 2 |
| G16319A | 1.6 | 16 |
| C16320T | 1.3 | 13 |
| T16324A | 0.1 | 1 |
| T16324C | 0.1 | 1 |
| T16325C | 1.6 | 16 |
| C16327T | 0.1 | 1 |
| T16342A | 0.1 | 1 |
| T16342G | 0.1 | 1 |
| A16343C | 0.2 | 2 |
| A16343G | 1.2 | 12 |
| C16344T | 0.3 | 3 |
| T16352C | 1.0 | 10 |
| C16354T | 0.3 | 3 |
| C16355T | 1.3 | 13 |
| T16356C | 3.7 | 37 |
| T16357C | 0.3 | 3 |
| C16360G | 0.1 | 1 |
| C16360T | 0.1 | 1 |
| T16362C | 6.9 | 69 |
| C16364A | 0.1 | 1 |
| C16365T | 0.1 | 1 |
| C16366T | 0.1 | 1 |
| T16368C | 0.2 | 2 |
| G16369C | 0.2 | 2 |
| G16370A | 0.2 | 2 |
| A16387G | 0.2 | 2 |
| G16390A | 1.7 | 17 |
| G16391A | 1.2 | 12 |
| T16396G | 0.2 | 2 |
| T16397G | 0.3 | 3 |
| G16398A | 0.5 | 5 |
| A16399G | 3.9 | 39 |
| C16400T | 0.2 | 2 |
| C16411A | 0.1 | 1 |
| G16428A | 0.2 | 2 |
| G16438A | 0.1 | 1 |
| C16439A | 0.3 | 3 |
| C16439G | 0.1 | 1 |
| A16463G | 0.8 | 8 |
| A16463T | 0.2 | 2 |
| C16465T | 0.1 | 1 |
| T16468G | 0.1 | 1 |
| G16474T | 0.1 | 1 |
| C16478A | 0.3 | 3 |
| A16482G | 1.8 | 18 |
| G16485A | 0.1 | 1 |
| A16486G | 0.1 | 1 |
| T16489A | 0.1 | 1 |
| T16489C | 0.1 | 1 |
| T16491C | 0.1 | 1 |
| C16495A | 0.2 | 2 |
| G16496A | 0.1 | 1 |
| T16519C | 66.8 | 670 |
| G16526A | 0.8 | 8 |
| G16526Del | 0.5 | 5 |
| C16528A | 0.1 | 1 |
| T10C | 0.1 | 1 |
| C11A^2^ | 0.1 | 1 |
| A16T | 0.1 | 1 |
| C19A^2^ | 0.2 | 2 |
| C44InsC | 0.2 | 2 |
| T46A^2^ | 0.1 | 1 |
| G54InsC | 0.4 | 4 |
| T55C | 0.1 | 1 |
| T55G | 0.4 | 4 |
| A56G | 0.4 | 4 |
| C64T | 0.2 | 2 |
| G71A | 0.2 | 2 |
| T72A | 0.1 | 1 |
| T72C | 2.5 | 25 |
| T72G | 0.2 | 2 |
| A73G | 53.9 | 541 |
| A93G | 0.6 | 6 |
| T119C | 0.2 | 2 |
| T131C | 0.1 | 1 |
| G143A | 0.9 | 9 |
| T146C | 9.2 | 92 |
| C150T | 10.6 | 106 |
| C151T | 1.0 | 10 |
| T152C | 22.8 | 229 |
| A153G | 1.9 | 19 |
| T155C | 0.2 | 2 |
| T158C^2^ | 0.1 | 1 |
| T159C | 0.1 | 1 |
| C182T | 0.5 | 5 |
| A183G | 0.7 | 7 |
| G185A | 5.5 | 55 |
| A188G | 0.9 | 9 |
| A189G | 4.5 | 45 |
| A193G | 0.4 | 4 |
| C194T | 2.0 | 20 |
| T195C | 16.9 | 170 |
| T196C | 0.4 | 4 |
| C198T | 0.2 | 2 |
| T199C | 3.2 | 32 |
| A200G | 1.8 | 18 |
| A201G | 0.1 | 1 |
| G203A | 0.1 | 1 |
| G203C | 0.1 | 1 |
| T204C | 4.8 | 48 |
| G207A | 3.3 | 33 |
| T212C | 0.1 | 1 |
| A214G | 0.1 | 1 |
| A214T | 0.1 | 1 |
| A215G | 2.5 | 25 |
| T217C | 1.5 | 15 |
| C222T | 0.2 | 2 |
| G225A | 1.8 | 18 |
| T226C | 0.6 | 6 |
| A227G | 0.5 | 5 |
| A227T | 0.2 | 2 |
| G228A | 5.3 | 53 |
| T236C | 0.3 | 3 |
| T239C | 2.0 | 20 |
| C242A^2^ | 0.1 | 1 |
| C242T | 1.4 | 14 |
| G247A | 0.1 | 1 |
| A249G | 0.3 | 3 |
| A249Del | 0.2 | 2 |
| T250C | 1.1 | 11 |
| A257G | 0.5 | 5 |
| C262T | 0.2 | 2 |
| A263G | 98.9 | 992 |
| A272G | 0.1 | 1 |
| T282C | 0.3 | 3 |
| C285T | 0.1 | 1 |
| A291C | 0.1 | 1 |
| T292C | 0.1 | 1 |
| T294C | 0.1 | 1 |
| T294Del^2^ | 0.1 | 1 |
| T294InsT | 0.3 | 3 |
| C295A | 0.1 | 1 |
| C295T | 10.1 | 101 |
| C296A^2^ | 0.1 | 1 |
| C296T | 0.2 | 2 |
| A297G | 0.4 | 4 |
| C299Del | 0.1 | 1 |
| A302C-Ins | 39.2 | 393 |
| A302CC-Ins | 11.7 | 117 |
| A302CCC-Ins | 0.5 | 5 |
| T310C-Ins | 97.3 | 976 |
| T310TC-Ins | 2.2 | 22 |
| T310TCC-Ins | 0.1 | 1 |
| G316A | 0.1 | 1 |
| T319C | 1.3 | 13 |
| C324T | 0.1 | 1 |
| C327T | 0.1 | 1 |
| A335G | 0.1 | 1 |
| C340T | 1.3 | 13 |
| C345T | 0.1 | 1 |
| A360C^2^ | 0.1 | 1 |
| A360G^2^ | 1.6 | 16 |
| G366A | 0.5 | 5 |
| A368G | 0.1 | 1 |
| A373G | 0.2 | 2 |
| A374G | 0.3 | 3 |
| A384G | 0.1 | 1 |
| A385G | 0.2 | 2 |
| T408A | 0.1 | 1 |
| A426T^2^ | 0.1 | 1 |
| A428C^2^ | 0.1 | 1 |
| T430C | 0.3 | 3 |
| T449C | 0.1 | 1 |
| T452C | 0.1 | 1 |
| T452Del | 0.1 | 1 |
| C456T | 2.9 | 29 |
| C456TT | 0.1 | 1 |
| T460C | 0.2 | 2 |
| C462T | 8.1 | 81 |
| A464C | 0.1 | 1 |
| T477C | 2.9 | 29 |
| T477G | 0.1 | 1 |
| T482A^2^ | 0.1 | 1 |
| T482C | 0.7 | 7 |
| T482G^2^ | 0.1 | 1 |
| T485C | 0.1 | 1 |
| C486A | 0.1 | 1 |
| C486T | 0.1 | 1 |
| A487C^2^ | 0.1 | 1 |
| A487T^2^ | 0.1 | 1 |
| T489C | 11.3 | 113 |
| T489G | 0.1 | 1 |
| C494Del | 0.7 | 7 |
| C495A^2^ | 0.1 | 1 |
| C497T | 3.6 | 36 |
| G499A | 2.7 | 27 |
| T507C | 0.1 | 1 |
| A508G | 1.5 | 15 |
| C509A^2^ | 0.1 | 1 |

^1^n = Number of individuals with the respective polymorphism

^2^Polymorphisms not listed in Genbank, MITOMAP, or Phylotree

**Table C.** General and biochemical characteristics of the study populations.

|  | STYJOBS/ |  | URSPRUNG^4^ | SAPHIR^5^ | | SAPHIR^5^ |
| --- | --- | --- | --- | --- | --- | --- |
| Mean ± SD^1^ | EDECTA^3^ |  |  | Obese | | Controls |
| Age (years) | 12.9 ± 3.1 (n=248) |  | 16.6 ± 1.7 (n=266) | 51.9 ± 6.0 (n=1003) | | 51.5 ± 6.2 (n=595) |
| BMI kg/m² | 30.3 ± 6.1 (n=248) |  | 20.9 ± 1.9 (n=266) | 29.1 ± 3.5 (n=1003) | | 22.9 ± 1.6 (n=595) |
| BMI sds | 5.88 ± 2.6 (n=248) |  | -0.02 ± 0.6 (n=266) | n.a.^2^ | | n.a.^2^ |
| Body weight (kg) | 77.3 ± 25.0 (n=248) |  | 63.8 ± 9.1 (n=266) | 86.6 ± 12.6 (n=1001) | | 68.2 ± 9.3 (n=594) |
| Body height (m) | 1.58 ± 0.1 (n=248) |  | 1.74 ± 0.1 (n=266) | 172.5 ± 8.7 (n=1001) | 172.0 ± 9.2 (n=594) | |
| Glucose (mmol/l) | 5.02 ± 0.6 (n=248) |  | 4.46 ± 0.5 (n=265) | 5.35 ± 1.2 (n=1000) | 4.95 ± 0.6 (n=595) | |
| Insulin (mU/l) | 24.78 ± 30.2 (n=245) |  | 8.68 ± 6.9 (n=264) | 8.96 ± 5.8 (n=1000) | 4.95 ± 2.4 (n=588) | |
| HOMA | 5.88 ± 8.22 (n=246) |  | 1.73 ± 1.5 (n=266) | 2.23 ± 1.8 (n=994) | 1.10 ± 0.6 (n=588) | |
| DBP (mmHg) | 68.7 ± 9.0 (n=241) |  | 81.5 ± 10.2 (n=266) | 83.1 ± 7.8 (n=933) | 79.7 ± 7.2 (n=547) | |
| SBP (mmHg) | 125.8 ± 15.6 (n=241) |  | 126.9 ± 12.9 (n=266) | 136.5 ± 12.7 (n=933) | 128.9 ± 11.8 (n=547) | |
| TG (mmol/l) | 1.41 ± 0.8 (n=246) |  | 0.90 ± 0.4 (n=265) | 1.59 ± 1.0 (n=1000) | 1.12 ± 0.8 (n=595) | |
| LDL cholesterol (mmol/l) | 2.69 ± 0.7 (n=233) |  | 2.24 ± 0.6 (n=263) | 3.82 ± 0.9 (n=1000) | 3.66 ± 1.0 (n=595) | |
| HDL cholesterol (mmol/l) | 1.12 ± 0.3 (n=242) |  | 1.47 ± 0.3 (n=264) | 1.45 ± 0.4 (n=1000) | 1.70 ± 0.4 (n=595) | |
|  |  |  |  |  |  | |

^1^SD: standard deviation

^2^n.a. not available

^3^Juvenile obesity cohort

^4^Juvenile control cohort

^5^Adult cohort

BMI: Body Mass Index; BMI sds: BMI standard deviation score; HOMA: Homeostasis Model Assessment; DBP: diastolic blood pressure; SBP: systolic blood pressure; TG: triglycerides; LDL: low-density lipoprotein; HDL: high-density lipoprotein.
